# The clinical management of hepatic sarcoidosis: A systematic review

**DOI:** 10.1002/jgh3.13076

**Published:** 2024-06-20

**Authors:** Ram Prasad Sinnanaidu, Vikneshwaran Chandra Kumar, Ranita Hisham Shunmugam, Sanjiv Mahadeva

**Affiliations:** ^1^ Gastroenterology Unit, Medical Department Universiti Malaya Medical Centre Kuala Lumpur Malaysia; ^2^ Hepatology Department Hospital Selayang Batu Caves Malaysia; ^3^ Department of Library & Information Science, Faculty of Arts & Social Sciences Universiti Malaya Kuala Lumpur Malaysia

**Keywords:** anti‐TNF, Ursodeoxycholic acid, azathioprine, corticosteroids, hepatic sarcoidosis, methotrexate

## Abstract

**Background:**

Hepatic sarcoidosis is an uncommon clinical condition in which clear recommendations are lacking in its treatment. We aimed to review systematically the literature on hepatic sarcoidosis treatment to guide clinicians.

**Methods:**

Using MEDLINE, PubMed, CINAHL, Cochrane Library, and Google Scholar databases, we searched original articles on clinical studies reporting the outcome of adult hepatic sarcoidosis patients following treatment with various pharmacological agents. The primary end point was focused on assessing symptomatic relief and biochemical improvement posttreatment.

**Results:**

Out of 614 retrieved references, 34 published studies were eligible, providing data for a total of 268 patients with hepatic sarcoidosis. First‐line therapy with corticosteroids alone was reported in 187 patients, whilst ursodeoxycholic acid (UDCA) was used in 40 patients. Symptomatic and biochemical responses were reported among 113(60.4%) and 80(42.8%) cases of corticosteroids respectively, whereas UDCA showed a complete response in 23(57.5%) patients. Second‐line therapy was used in steroid‐refractory cases, with most cases being reported for azathioprine (*n* = 32) and methotrexate (*n* = 28). Notably, 15(46.9%) and 11(39.2%) patients showed both clinical and biochemical responses respectively. Biological therapy including anti‐tumor necrosis factor (anti‐TNF) was used as third line therapy in twelve cases with a 72.7% symptomatic and biochemical response rate each.

**Conclusion:**

The quality of evidence for the treatment of hepatic sarcoidosis was poor. Nevertheless, it appears that corticosteroid or UDCA may be utilized as first‐line therapy. For cases that are refractory to corticosteroids, steroid‐sparing immunosuppressive agents and anti‐TNF have shown some promising results, but further high‐quality studies are required.

## Graphical abstract

A systematic review of 34 published studies (*n* = 268) for the treatment of hepatic sarcoidosis revealed a 42.7% ‐ 60.4% clinical response rate for 1^st^‐line therapy (corticosteroids or UDCA), and a 39.2% ‐ 72.7% clinical response rate for 2^nd^‐line therapeutic agents (azathioprine, methotrexate and anti‐TNF). Further high‐quality studies are required to determine the efficacy of these treatments.

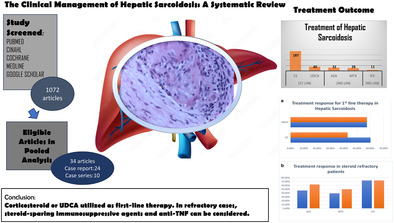

## Introduction

Sarcoidosis is a rare multisystem disorder of unknown etiology. It is characterized by the presence of noncaseating granuloma histologically. It primarily targets the lungs and hilar lymph nodes.^1^, ^2^, ^3^ Extrapulmonary diseases are often seen as part of systemic sarcoidosis with involvement of most organs particularly skin, eyes, liver, spleen, cardiac, salivary gland, and bone marrow.^4^ Although hepatic involvement in systemic sarcoidosis has been reported in 50–80% of autopsy studies,^2^, ^4^, ^5^, ^6^ isolated hepatic sarcoid is rare. Furthermore, most patients with hepatic sarcoidosis are asymptomatic or have mild disease,^2^, ^7^, ^8^ less than 1% of patients progress to severe cholestatic jaundice, portal hypertension, Budd‐Chiari syndrome, cirrhosis as well as end‐stage liver disease which requires liver transplant.^5^, ^8^, ^9^

There has been considerable progress in basic research and the diagnosis of hepatic sarcoidosis over the past few years. However, the treatment of hepatic sarcoidosis is still not well established.^5^, ^9^, ^10^, ^11^ Corticosteroids remain the mainstay of treatment despite a lack of prospective controlled studies.^6^ Failure or adverse effects of corticosteroids leads to the initiation of various other treatments as second‐line therapy to achieve remission, namely methotrexate, azathioprine, and tumor necrosis factor (TNF) inhibitors such as infliximab.^2^, ^5^, ^6^, ^9^, ^12^ However, the evidence for the timing and the choice of second‐line therapy is lacking.

In lieu of the ambiguity of the management of hepatic sarcoidosis, we conducted a systematic review of the literature on pharmacological therapy in hepatic sarcoidosis. The aim of this study was to identify available treatment strategies for patients with hepatic sarcoidosis, the appropriate timing to initiate treatment and to summarize their efficacy and outcomes.

## Methodology

### Protocol

**Figure 1:**
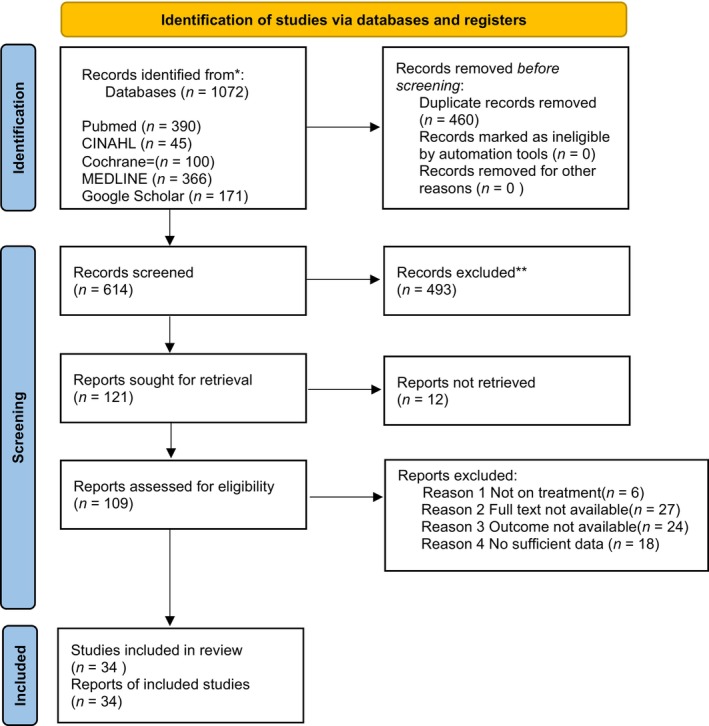
PRISMA flow diagram for the literature search in this study. *From*: Page MJ, McKenzie JE, Bossuyt PM, Boutron I, Hoffmann TC, Mulrow CD, et al. The PRISMA 2020 statement: an updated guideline for reporting systematic reviews. *BMJ* 2021;372:n71. doi: 10.1136/bmj.n71. For more information, visit: http://www.prisma‐statement.org/.

This protocol is registered with PROSPERO (ID: CRD42022350517). We have also adhered to the Preferred Reporting Items for Systematic Reviews and Meta‐Analyses (PRISMA) guideline^13^ on conducting and reporting this systematic review and meta‐analysis result as stated in Figure 1.

### Search strategy

We performed an electronic literature search in MEDLINE, PubMed, CINAHL, Cochrane Library, and Google Scholar, from inception to 31 July 2022. A combination of Medical Subject Headings and free text terms were used for the keywords ‘Hepatic sarcoidosis’ and ‘Corticosteroid**’** (see full search strategy for PubMed in Appendix I). These keywords had to appear in conjunction with the title, abstract, or full text of the article.

### Selection process

Following the search, all identified records were loaded into EndNote X20 (Clarivate Analytics, Philadelphia, PA, USA) and duplicates were removed. Two reviewers (RP and VK) worked in pairs to independently screen titles and abstracts, followed by full‐text papers using the inclusion and exclusion criteria. We included all studies with the following inclusion criteria: (a) any clinical study design reporting the outcome of human adult hepatic sarcoidosis patients, that is, case reports, case series, case–control studies or randomized clinical trials, (b) human adult hepatic sarcoidosis cases confirmed by histology/ liver biopsy, (c) intervention with any pharmacological agent, (d) either clinical symptoms, biochemical or histological response to treatment. There were no language restrictions and the only exclusion criteria were animal studies. Disagreements were resolved by discussion with the review team (SM) as necessary.

### Outcome measures

The primary end point of this systematic review was focused on assessing symptomatic relief and biochemical improvement posttreatment. All patients that been initiated on treatment will be evaluated base on symptom resolution and improvement in liver function test. With regard to biochemical improvement, we defined treatment response as follows:

#### Complete response

It was defined as the normalization of liver parameters at the end of treatment.

#### Partial response

Biochemical improvement in liver indices but without liver enzymes falling within the normal range was observed.

## Results

### Description of selected studies

A total of 614 references were retrieved from PubMed, Embase, and Cochrane Library databases. After abstract review and full‐text assessment, 34 published studies were selected (Fig. 1). Authors, study design, diagnostic criteria, inclusion and exclusion criteria, and sample size are summarized in Table 1. Notably, 24 (70%) of the selected studies were case reports, whilst 10 were case series. No prospective studies, nor randomized control trials were retrieved.

**Table 1:** Summary of clinical reports included in the systematic review

First author	Year of publications	Countries	Number of centers	Study design	Inclusion criteria	Sample size	References number
Abid	2019	USA	Single center	Case report	Patient diagnosed with HS	1	^36^
Bakker	2012	Netherlands	Single center	Case series	Patient diagnosed with HS	17	^22^
Coelho	2018	USA	Single center	Case report	Patient diagnosed with HS	1	^37^
De Mulder	2019	Belgium	Single center	Case report	Patient diagnosed with HS	1	^38^
Deliwala	2021	USA	Single center	Case report	Patient diagnosed with HS	1	^52^
Doty	2005	USA	Single center	Case series	Patient diagnosed with HS	10	^14^
Ennaifer	2016	Tunisia	Single center	Case series	Patient diagnosed with HS	7	^21^
Farouj	2011	France	Single center	Case report	Patient diagnosed with HS	1	^45^
Ghoneim	2019	USA	Single center	Case series	Patient diagnosed with HS	3	^26^
Graf	2021	German	Multi center^5^	Case series	Patient diagnosed with HS	62	^20^
Harder	2007	German	Single center	Case report	Patient diagnosed with HS	1	^46^
Ibrahim	2018	USA	Single center	Case report	Patient diagnosed with HS	1	^39^
Israel	1984	USA	Single center	Case series	Patient diagnosed with HS	15	^25^
Jovicić	2014	Serbia	Single center	Case report	Patient diagnosed with HS	1	^40^
Kennedy	2006	United Kingdom	Multi center	Case series	Patient diagnosed with HS	180	^8^
Kothakota	2021	India	Single center	Case report	Patient diagnosed with HS	1	^50^
Kundu	2004	United Kingdom	Single center	Case report	Patient diagnosed with HS	1	^29^
Malhotra	2008	USA	Single center	Case report	Patient diagnosed with HS	1	^23^
Masuda K	2018	Japan	Single center	Case report	Patient diagnosed with HS	1	^44^
Melissant	1993	Netherland	Single center	Case report	Patient diagnosed with HS	1	^41^
Mosea	2011	United Kingdon	Multi center	Case series	Patient diagnosed with HS	3	^24^
Mueller	2000	German	Single center	Case report	Patient diagnosed with HS	1	^48^
Park	2022	Korea	Single center	Case report	Patient diagnosed with HS	1	^15^
Rawala	2020	USA	Single center	Case report	Patient diagnosed with HS	1	^34^
Rezeig	1997	Saudi Arabia	Single center	Case report	Patient diagnosed with HS	1	^42^
Sedki	2019	USA	Single center	Case series	Patient diagnosed with HS	27	^11^
Sharma	2006	USA	Single center	Case report	Patient diagnosed with HS	1	^49^
Stitt	2014	USA	Single center	Case report	Patient diagnosed with HS	1	^43^
Tan	2012	USA	Single center	Case report	Patient diagnosed with HS	1	^47^
Tasbakan	2014	Turkey	Single center	Case report	Patient diagnosed with HS	1	^16^
Ungprasert	2017	USA	Single center	Case series	Patient diagnosed with HS	19	^6^
Watanabe	2018	Japan	Single center	Case report	Patient diagnosed with HS	1	^17^
Ying C	2021	USA	Single center	Case report	Patient diagnosed with HS	1	^19^
Yu K–K	2015	China	Single center	Case report	Patient diagnosed with HS	1	^18^

### Quantitative analysis

#### Baseline characteristics

Main baseline patient characteristics, including average age, ethnicity, clinical outcomes, and treatment, have been summarized in Table 2. The selected studies included 273 patients diagnosed either as primary disease or extra manifestation of other primary organ involvement. A bimodal pattern was observed with early peak at the age of 20–40 years and a later peak at 50–70 years old. The follow‐up ranged from 2 months to a period of 11 years in one study. The most common symptom reported was abdominal pain, particularly over the right upper quadrant, stated in 15 studies. This was followed by weight loss, reported in 14 studies. Fever, fatigue, and jaundice were equally reported in nine studies. Isolated hepatic sarcoidosis was discussed only in five studies. Pulmonary disease was the commonest extrahepatic manifestation, reported in 18 studies, followed by ocular (8), lymphatic (6), cutaneous (6), spleen (5), and cardiac (5).

**Table 2:** Summary of treatment and outcomes of patients with hepatic sarcoidosis from clinical reports

First author	Year of publication	Sample size	Gender	Average Age	Mean follow‐up (month)	Number of patients treated with steroids	Number of patients treated with steroids plus immunosuppressant	Immunosuppressor used	Treatment response (%) (Biochemically)
Abid^1^	2019	1	F	37	NA	1	0		100
Bakker^17^	2012	17	M: 10 F: 7	37–77	3	3	0		66
Coelho^1^	2018	1	F	51	6	1	0		100
De Mulder^1^	2019	1	M	68	2	1	0		100
Deliwala^1^	2021	1	M	47	3	1	0		100
Doty^1^	2005	10	M:1 F: 9	16–44	2	1	1	AZA/ Pentoxifylline/ Infliximab	100
Ennaifer^7^	2016	7	M:2 F: 5	31–59	12	5	0		60
Farouj^1^	2011	1	F	54	7	1	0		100
Ghoneim^3^	2019	3	M	31–59	NA	3	1	MTX	33
Graf (62)	2021	62	M:30 F: 32	51.6 ± 12.1	NA	43	23	AZA/MTX/MMF/Infliximab	60
Harder^1^	2007	1	M	71	NA	1	0		100
Ibrahim^1^	2018	1	F	68	1	1	0		100
Israel^15^	1984	15	M:3 F: 12	14–69	15–132	14	2	MTX/ Chlorambucil	100
Jovicic^1^	2014	1	M	69	18	1	0		100
Kennedy(90)	2006	180	M:91 F: 89	17–72	24	63	16	AZA/MTX	73
Kothakota^1^	2021	1	F	56	3	1	0		100
Kundu^1^	2004	1	F	34	NA	1	0		100
Malhotra^1^	2008	1	M	29	12	1	1	AZA	100
Masuda K^1^	2018		F	68	12	1	0		100
Melissant^1^	1993	1	M	58	12	1	0		100
Mosea^3^	2011	3	M:1 F: 2	40–75	24–60	3	2	AZA/MMF	100
Mueller^1^	2000	1	M	59	24	1	0		100
Park^1^	2022	1	M	37	49	1	1	AZA	100
Rawala^1^	2020	1	F	39	3	1	0		100
Rezeig^1^	1997	1	M	43	NA	1	0		100
Sedki^27^	2019	27	M:7 F: 20	45.7 (median)	84	17	15	AZA/MTX/Infliximab	88
Sharma^1^	2006	1	F	54	24	1	0		100
Stitt^1^	2014	1	F	42	NA	1	0		100
Tan^1^	2012		M	48	12	1	0		0
Tasbakan^1^	2014		F	F	15	1	0		100
Ungprasert^19^	2017		M:9 F:10	46.1 (mean age)	NA	11	4	MTX/HCQ/Infliximab	100
Watanabe^1^	2018		F	41	24	1	1	Adalimumab	100
Ying C^1^	2021		M	29	6	1	0		100
Yu K–K^1^	2015		F	50	12	1	0		100

#### Treatment regimens

##### First‐line therapy

Among 268 patients, 187 were treated with corticosteroids as first‐line therapy. Ursodeoxycholic acid (UDCA) had additionally been administered in 40 patients as first‐line therapy either as a single agent or in combination with corticosteroids. Both regimens are listed in Table 2. The most common type of corticosteroid used was prednisolone within a dose ranging from 20 to 60 mg/day, tapered every 6–8 weeks over a 6‐month period. Apart from prednisolone, methylprednisolone had been used in five studies with a dose range between 16 mg/day and 1 g/day.^14^, ^15^, ^16^, ^17^, ^18^ In one study, budesonide with a dosing of 3 mg twice daily was used. However, data on the duration of maintenance doses were unavailable.^19^ With regard to UDCA, the dosage that was prescribed ranged from 10 to 15 mg/kg or 300 mg twice daily.

For symptom improvement, 113 (60.2%) showed symptom resolution post‐treatment with corticosteroids in 23 studies. A partial response or no response was shown for corticosteroids in the remaining 10 studies (*n* = 74, 39.8%). With regard to biochemical response, 80 (43%) patients showed significant improvement posttreatment with corticosteroids in 18 studies. There was either a partial (*n* = 11, 5.9%) or no response (*n* = 57, 30.6%) in the remaining 12 studies. UDCA had shown a complete response (clinically and biochemically) in 23 (57.5%) patients, in five out of eight studies.^15^, ^20^, ^21^ Bakker et al. in fact reported that UDCA was superior to corticosteroids in treatment response, especially with regard to biochemical resolution.^22^

##### Second‐line therapy

The steroid‐sparing immunosuppressive agents used as second‐ or third‐line therapy included azathioprine, methotrexate, mycophenolate mofetil, cyclophosphamide, chlorambucil, infliximab and adalimumab. The number of cases utilizing second‐line agents were as follows: azathioprine *n* = 32, methotrexate *n* = 28, mycophenolate mofetil *n* = 2, cyclophosphamide *n* = 2 and chlorambucil *n* = 1. None of the studies reported in detail regarding the dosage of immunosuppressive agents. Only one study suggested that the dose of azathioprine should be initiated at 25 mg /day and to adjust accordingly based on response.

Second‐line therapy was usually initiated due to failure of first‐line treatment or as a steroid‐refractory agent. Among six studies which utilized azathioprine, a total of 20(62.5%) patients showed either clinical or biochemical improvement. Both biochemical and symptomatic improvement were observed in 15 patients (five studies),^8^, ^11^, ^15^, ^23^, ^24^ including histological improvement in one study^24^ and two studies only showed biochemical resolution.^15^, ^20^ No improvement with azathioprine was only shown in one study (*n* = 1, 3.1%).^14^

Methotrexate was the next most common second‐line agent utilized in four studies. Clinical and biochemical improvement was reported in three studies (*n* = 11.,39.2%),^8^, ^11^, ^25^ whilst one study (*n* = 3, 10.7%) only had biochemistry resolution.^20^ Progression to cirrhosis was reported in a single case report, despite on both prednisolone and methotrexate therapy.^26^

Both AZA and MTX have the potential for drug‐induced liver injury (DILI). However, the rate of DILI appeared to be low with only two cases reported by Graf et al., whereby AZA and MTX had to be discontinued due to hepatotoxicity effect.^20^

Other steroid‐sparing immunosuppressive therapy that have been reported in hepatic sarcoidosis include mycophenolate mofetil [MMF] and chlorambucil, but the case numbers were low. MMF was administered in only two patients in two different studies after a failure of corticosteroids, with improvement in biochemistry and symptoms.^20^, ^24^ Similarly, Chlorambucil resulted in the resolution of symptoms and liver function in a single case reported by Israel.^25^

##### Third‐line therapy

The number of cases utilizing third line agents were as follows: Anti‐tumor necrosis factor (anti‐TNF) (Infliximab) *n* = 11, Adalimumab *n* = 1. Infliximab was started in two patients at a dose of 5 mg/kg @ 0, 2,6 weeks then at every 6 week interval.^14^, ^20^ 8 patients on Infliximab have showed both clinical and biochemical improvement.

In a single case report by Watanabe et al., adalimumab was added following a partial response to corticosteroids. There was biochemical improvement with normalization of liver function and significant improvement on imaging. This effect continued for up to 2 years of follow‐up.

**Figure 2:**
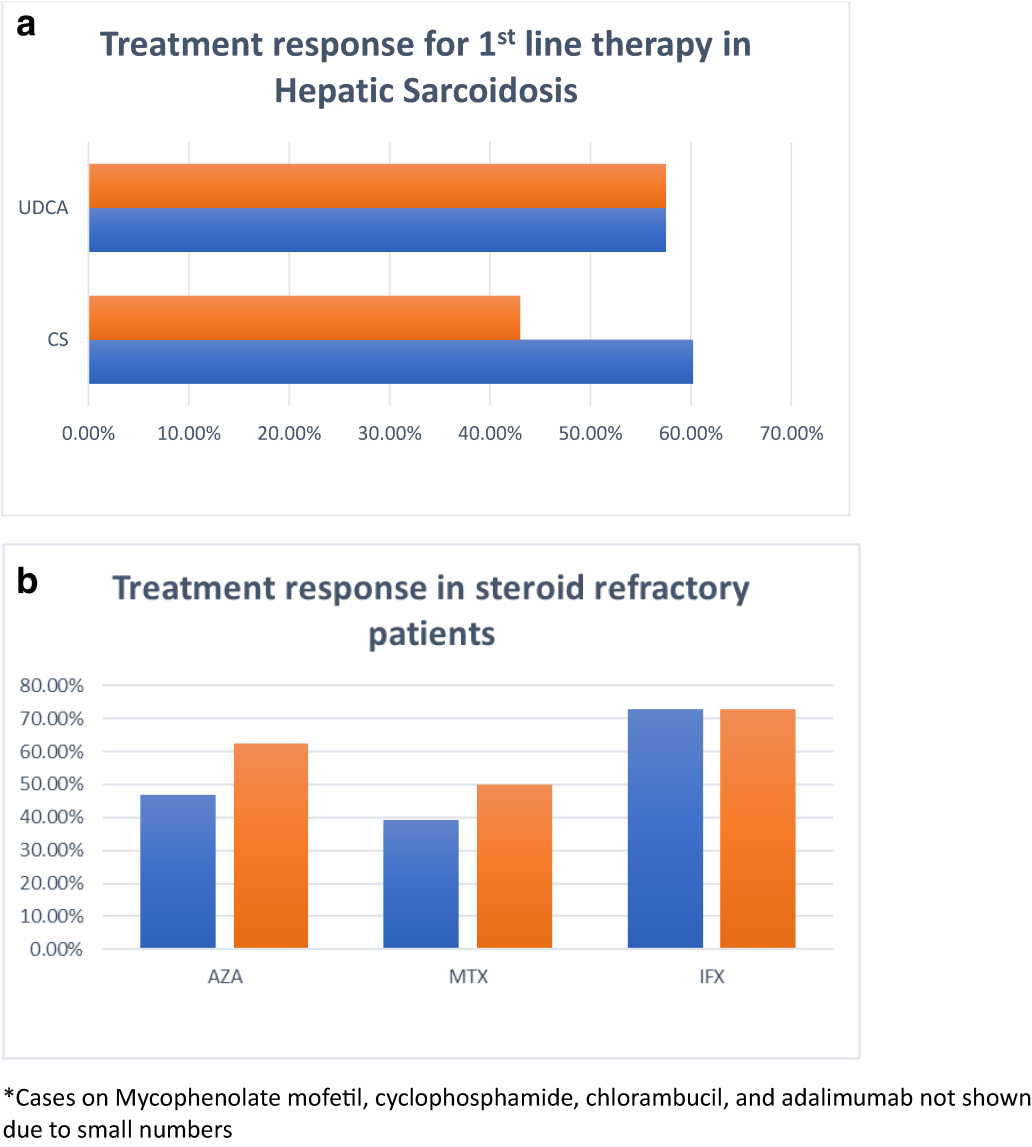
Graphical summary of treatment efficacy for various drugs in hepatic sarcoidosis. *Cases on Mycophenolate mofetil, cyclophosphamide, chlorambucil, and adalimumab not shown due to small numbers. (a) 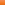 biochemical improvement, 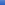 symptomatic improvement. Abbreviations: CS, corticosteroids; UDCA, ursodeoxycholic acid. (b) 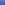 symptomatic improvement, 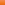 biochemical improvement. Abbreviations: AZA, azathioprine; IFX, infliximab; MTX, methotrexate.

Figure 2 Graphical summary of treatment efficacy for various drugs in hepatic sarcoidosis.

## Discussion

This systematic review has highlighted that most of the available published data on the treatment of hepatic sarcoid have been based on case reports, case series, expert opinions and extrapolation from the pulmonary sarcoidosis literature. Despite this low quality evidence data, it can be summarized that corticosteroids are the main first‐line treatment in hepatic sarcoidosis. The indication for therapy has been based on the presence of symptoms and biochemical liver derangement, with or without evidence of fibrosis/ cirrhosis on imaging.^9^, ^27^, ^28^, ^29^, ^30^, ^31^, ^32^, ^33^, ^34^, ^35^, ^36^ The dosage of corticosteroids, particularly prednisolone, varies from 20 mg to 60 mg per day.^12^, ^27^, ^37^, ^38^, ^39^, ^40^, ^41^, ^42^, ^43^, ^44^ In our review, some of the studies used dosages of 0.5 mg to 1 mg/kg/day,^45^, ^46^, ^47^ and none of the studies reported a prednisolone dose of greater than 1 mg/kg/day. Two review articles suggested a time frame of 4–6 weeks for initial duration of treatment, and subsequently to taper off prednisolone 5‐10 mg every 4–8 weeks.^5^, ^12^, ^48^, ^49^ The lowest effective steroid dose was maintained for long‐term treatment in most studies,^48^ provided there was a good initial response. Despite its efficacy, the prolonged use of corticosteroids has to be balanced with well‐known adverse effects of weight gain, hyperglycaemia, and increased risk of infections, gastrointestinal bleeding, and metabolic bone disease.^9^, ^50^

A few studies have shown that UDCA may be effective in hepatic sarcoidosis, but mainly in patients with cholestasis or a predominant symptom of pruritus. The recommended dosage of 10 mg/kg/day used in most of the reported studies^27^, ^30^, ^51^, ^52^ demonstrated improvement in liver biochemistry and symptoms of cholestasis, particularly pruritus.^12^, ^45^, ^51^ Although the patients included in this systematic review did not undergo repeated biopsy posttreatment, it has been suggested that UDCA does not impede histological progression of the disease.^9^, ^27^

For cases which were refractory to corticosteroids or needed longer term immunosuppression, the available studies appear to favor azathioprine mostly, followed by methotrexate.

However, the potential risk of hepatotoxicity from MTX may limit its utility in hepatic sarcoidosis.^53^ The efficacy for other immune‐suppressants such as MMF and chlorambucil have only been reported in very small number of patients (*n* < 5) and hence remain uncertain.

Tumor necrosis factor (TNF) alpha is implicated in the pathogenesis of sarcoidosis by inducing inflammation and granuloma formation.^54^ Thus, TNF inhibitors may prevent granuloma pathogenesis by binding to TNF‐alpha and inhibiting its activity.^5^, ^55^ In this review, the few studies exploring anti‐TNF, which includes infliximab or adalimumab therapy, have suggested that they may be helpful in liver biochemistry improvement, but the small number of cases (*n* = 11) was not sufficient to demonstrate a significant improvement.

This systematic review has highlighted the lack of good quality evidence for the treatment of hepatic sarcoidosis. There were no randomized controlled trials nor prospective studies which have been published to date. Most of the data available were from case reports or case series, which might have led to a publication bias. However, due to the lack of randomized controlled trials nor prospective studies in this area, we have decided to include such studies. Another limitation was the heterogeneity of the endpoints, which did not allow for comparison between outcomes. There is no standardized method to evaluate outcomes, such as posttreatment liver biopsy, as well as a standardized definition to conclude treatment response. Furthermore, the duration of treatment varied widely from one study to another. Moreover, data on adverse drug events were not provided in all studies, making safety comparisons between corticosteroids and steroid‐sparing immunosuppressants difficult. Lastly, there may have been some publication bias as some cases of isolated hepatic sarcoidosis do not develop symptoms nor serious sequelae.

**Figure 3:**
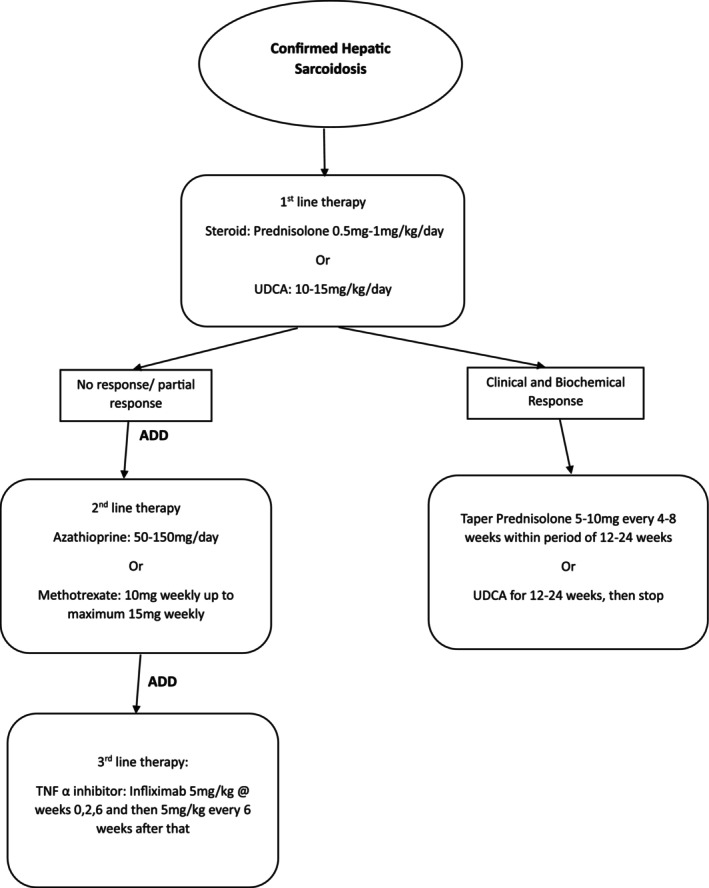
Proposed treatment algorithm for hepatic sarcoidosis, based on available data from the current literature.

Taking into consideration of these limitations in the context of a rare disease, with the available data, we suggest the following as a reasonable approach to the treatment of hepatic sarcoidosis: an early corticosteroid treatment at 0.5 to 1 mg/kg/day with a 3 to 6 months tapering scheme in cases of clinical and biochemical/ imaging remission, and an adjunctive therapy by a steroid sparing agent such as AZA and MTX at usual doses. (Fig. 3).

Patients' follow‐up should be based on their initial presentation (evidence of cirrhosis, fibrosis, or liver failure). Despite the interest in TNF‐α antagonists as a potential treatment modality in hepatic sarcoidosis, the published evidence in the literature remains limited. We conclude that further studies with homogenous groups, comparisons between the different treatments' schemes and with reproducible strong endpoints are needed.

## Data Availability

The datasets obtained and/or analyzed during the current study are available from the corresponding author on reasonable request.

## Appendix I

**Table:**

No	Database	Search strategy	Hits
1	25/7/2022 Pubmed	(((((“hepatic sarcoid*”[Title/Abstract]) OR (“hepatic granuloma”[Title/Abstract])) OR (“extrapulmonary sarcoid*”[Title/Abstract])) OR (“Liver granuloma”[Title/Abstract])) OR (“liver sarcoid*”[Title/Abstract])) AND (((Corticosteroid[Title/Abstract] OR Glucocorticoid[Title/Abstract] OR Prednisone[Title/Abstract] OR prednisolone[Title/Abstract] OR “Immunosuppressive therapy”[Title/Abstract] OR Steroid[Title/Abstract] OR methotrexate[Title/Abstract] OR azathioprine[Title/Abstract] OR immunomodulators[Title/Abstract]) OR (“Ursodeoxycholic acid”[Title/Abstract] OR Manag*[Title/Abstract] OR Approach*[Title/Abstract] OR Treat*[Title/Abstract] OR Therap*[Title/Abstract] OR method*[Title/Abstract] OR cure[Title/Abstract] OR “liver transplant*”[Title/Abstract])) OR ((((((((((“Glucocorticoids/therapeutic use”[Mesh]) OR ((“Prednisone/therapeutic use”[Mesh] OR “Prednisone/therapy”[Mesh]))) OR ((“Immunosuppression Therapy/therapeutic use”[Mesh] OR “Immunosuppression Therapy/therapy”[Mesh]))) OR ((“Steroids/therapeutic use”[Mesh] OR “Steroids/therapy”[Mesh]))) OR (“Methotrexate/therapeutic use”[Mesh])) OR (“Azathioprine/therapeutic use”[Mesh])) OR (“Infliximab/therapeutic use”[Mesh])) OR (“Ursodeoxycholic Acid/therapeutic use”[Mesh])) OR (“Liver Transplantation/therapy”[Mesh])) OR ((“Adrenal Cortex Hormones/therapeutic use”[Mesh]) OR (“Adrenal Cortex Hormones/therapy”[Mesh]))))	390

## References

[1] Culver DA . Sarcoidosis. Immunol. Allergy Clin. North Am. 2012; 32: 487–511. 23102063 10.1016/j.iac.2012.08.005

[2] Tadros M , Forouhar F , Wu GY . Hepatic sarcoidosis. J. Clin. Transl. Hepatol. 2013; 1: 87–93. 26357609 10.14218/JCTH.2013.00016 PMC4521279

[3] Crouser ED , Maier LA , Wilson KC *et al*. Diagnosis and detection of sarcoidosis. An official american thoracic society clinical practice guideline. Am. J. Respir. Crit. Care Med. 2020; 201: e26–e51. 32293205 10.1164/rccm.202002-0251ST PMC7159433

[4] Ebert EC , Kierson M , Hagspiel KD . Gastrointestinal and hepatic manifestations of sarcoidosis. Am. J. Gastroenterol. 2008; 103: 3184–3192 quiz 93. 18853979 10.1111/j.1572-0241.2008.02202.x

[5] Deutsch‐Link S , Fortuna D , Weinberg EM . A comprehensive review of hepatic sarcoid. In: Seminars in Liver Disease. Thieme Medical Publishers, New York; 2018. 10.1055/s-0038-1666853 30041280

[6] Ungprasert P , Crowson CS , Simonetto DA , Matteson EL . Clinical characteristics and outcome of hepatic sarcoidosis: a population‐based study 1976–2013. Am. J. Gastroenterol. 2017; 112: 1556–1563. 28872150 10.1038/ajg.2017.231 PMC5629110

[7] Iannuzzi MC, Rybicki BA, Teirstein AS. Sarcoidosis. N Engl J Med. 2007; 357: 2153–2165. 18032765 10.1056/NEJMra071714

[8] Kennedy PT , Zakaria N , Modawi SB *et al*. Natural history of hepatic sarcoidosis and its response to treatment. Eur. J. Gastroenterol. Hepatol. 2006; 18: 721–726. 16772828 10.1097/01.meg.0000223911.85739.38

[9] Ayyala US , Padilla ML . Diagnosis and treatment of hepatic sarcoidosis. Curr. Treat. Options Gastroenterol. 2006; 9: 475–483. 17081481 10.1007/s11938-006-0004-9

[10] Martin‐Blondel G , Camara B , Selves J *et al*. Etiology and outcome of liver granulomatosis: a retrospective study of 21 cases. Rev. Med. Interne. 2010; 31: 97–106. 19962798 10.1016/j.revmed.2009.10.430

[11] Sedki M , Fonseca N , Santiago P *et al*. Hepatic sarcoidosis: natural history and management implications. Front. Med. (Lausanne). 2019; 6: 232. 31737633 10.3389/fmed.2019.00232 PMC6831521

[12] Cremers JP , Drent M , Baughman RP , Wijnen PA , Koek GH . Therapeutic approach of hepatic sarcoidosis. Curr. Opin. Pulm. Med. 2012; 18: 472–482. 22617809 10.1097/MCP.0b013e3283541626

[13] Page MJ , McKenzie JE , Bossuyt PM *et al*. The PRISMA 2020 statement: an updated guideline for reporting systematic reviews. Syst. Rev. 2021; 10: 89. 33781348 10.1186/s13643-021-01626-4 PMC8008539

[14] Doty JD , Mazur JE , Judson MA . Treatment of sarcoidosis with infliximab. Chest. 2005; 127: 1064–1071. 15764796 10.1378/chest.127.3.1064

[15] Park YJ , Woo HY , Kim MB , Ahn J , Heo J . Primary hepatic sarcoidosis presenting with cholestatic liver disease and mimicking primary biliary cholangitis: a case report. J. Yeungnam Med. Sci. 2022; 39: 256–261. 34411476 10.12701/yujm.2021.01151 PMC9273146

[16] Taşbakan M , Erdem H , Pullukçu H *et al*. Isolated hepatic sarcoidosis mimicking liver microabscesses: a case report. Ir. J. Med. Sci. (1971). 2014; 183: 503–505. 10.1007/s11845-014-1074-7 24563258

[17] Watanabe T , Jodo S . Hepatic sarcoidosis. Cmaj. 2018; 190: E988. 30127039 10.1503/cmaj.180276 PMC6102111

[18] Yu K‐K , Liu H‐Q , Zhou Z‐W , Chen M‐Q . Hepatic sarcoidosis mimicking liver cancer. Int. J. Clin. Exp. Med. 2015; 8: 9607–9609. 26309634 PMC4538051

[19] Ying C , Ciobanu C , Mohrmann L . Hepatic sarcoidosis resembling primary sclerosing cholangitis. BMJ Case Rep. 2021; 14: 1–4. 10.1136/bcr-2021-243492 PMC8424844 34493554

[20] Graf C , Arncken J , Lange CM *et al*. Hepatic sarcoidosis: Clinical characteristics and outcome. JHEP Rep. 2021; 3: 100360. 34765958 10.1016/j.jhepr.2021.100360 PMC8571721

[21] Ennaifer R , Ayadi S , Romdhane H *et al*. Hepatic sarcoidosis: a case series. Pan Afr. Med. J. 2016; 24: 209. 27795804 10.11604/pamj.2016.24.209.7980 PMC5072846

[22] Bakker GJ , Haan YC , de Buy M , Wenniger LJ , Beuers U . Sarcoidosis of the liver: to treat or not to treat? Neth. J. Med. 2012; 70: 349–356. 23065982

[23] Malhotra A , Naniwadekar A , Sood G . Hepatobiliary and pancreatic: cirrhosis secondary to hepatic sarcoidosis. J. Gastroenterol. Hepatol. 2008; 23: 1942. 19120880 10.1111/j.1440-1746.2008.05707.x

[24] Mosea H , Gotto J , Khan Z . Diagnostic and therapeutic challenges of hepatic sarcoidosis. BMJ Case Rep. 2011; 2011: bcr0420114069. 10.1136/bcr.04.2011.4069 PMC3139159 22689553

[25] Israel HL , Margolis ML , Rose LJ . Hepatic granulomatosis and sarcoidosis. Further observations. Dig. Dis. Sci. 1984; 29: 353–356. 6705647 10.1007/BF01318522

[26] Ghoneim S , Williams SD . Hepatic sarcoidosis: an uncommon cause of cirrhosis. Cureus. 2019; 11: e6316. 10.7759/cureus.6316 PMC6901370 31857928

[27] Baughman R , Bari K . Gastrointestinal Involvement of Sarcoidosis. In: Handbook of Systemic Autoimmune Diseases, Vol. 13. Elsevier, Amsterdam, The Netherlands, 2017; 377–397.

[28] Hon SA , Lee L , Tan K , Lee R , Low Q . Hepatic sarcoidosis: diagnostic approach and management. Med. J. Malaysia. 2021; 76: 914–917. 34806684

[29] Kundu S , Gautam M , Jain H , Shameem M . Hepatic sarcoidosis causing intractable pruritis: 590. Official J. Am. College Gastroenterol. 2004; 99: S191.

[30] Miyamoto R , Sano N , Tadano S , Inagawa S , Adachi S , Yamamoto M . Hepatic sarcoidosis mimicking cholangiocellular carcinoma: A case report and literature review. Int. J. Surg. Case Rep. 2017; 41: 165–168. 29080443 10.1016/j.ijscr.2017.10.032 PMC5686224

[31] Morris C , Drinkwater K , Varshney N . Hepatic sarcoidosis presenting as hypercalcemia. Am. J. Med. 2020; 133: e727–e728. 32603790 10.1016/j.amjmed.2020.05.031

[32] Muhanna A , Al Momani L , Likhitsup A . Sarcoidosis manifesting as liver granuloma with asteroid bodies. Cureus. 2021; 13: e17915. 34540506 10.7759/cureus.17915 PMC8439400

[33] Nguyen V , Ngo H , Ngo HN , Awad HH . Spontaneous resolution of symptomatic hepatic sarcoidosis. Case Rep. Gastrointest. Med. 2018; 2018: 1535049. 30155317 10.1155/2018/1535049 PMC6093030

[34] Rawala MS , Ahmed AS , Helmick K . An atypical presentation of extrapulmonary sarcoidosis. Case Rep. Rheumatol. 2020; 2020: 8840245. 32670655 10.1155/2020/8840245 PMC7334768

[35] Inoue M , Chiba T , Zen Y *et al*. Hepatic sarcoidosis with an increased serum level of immunoglobulin G4. Intern. Med. 2012; 51: 3095–3098. 23124158 10.2169/internalmedicine.51.8224

[36] Abid H , Siddiqui N . A rare case of necrotizing sarcoid granulomatosis involving liver. Cureus. 2019; 11: e5366. 10.7759/cureus.5366 PMC6783217 31608200

[37] Coelho‐Prabhu N , Kamath P . Resolution of portal hypertension following steroid therapy for hepatic sarcoidosis: 654. Official J. Am. College Gastroenterol. 2008; 103: S254–S255.

[38] De Mulder P , Maertens B , Hoorens A , Vonck A . Extrapulmonary sarcoidosis primarily presenting as cholestatic liver disease. BMJ Case Rep. 2019; 12: 1–5. 10.1136/bcr-2019-232618 PMC6904153 31806633

[39] Ibrahim AM , Bhandari B , Soriano PK *et al*. Hepatic involvement in systemic sarcoidosis. Am. J. Case Rep. 2018; 19: 1212–1215. 30305603 10.12659/AJCR.910600 PMC6196583

[40] Jovicić I , Popović D , Toncev L *et al*. Isolated hepatic sarcoidosis. Vojnosanit. Pregl. 2014; 71: 399–403. 24783422 10.2298/vsp1404399j

[41] Melissant CF , Smith SJ , Kazzaz BA , Demedts M . Bleeding varices due to portal hypertension in sarcoidosis. Favorable effect of propranolol and prednisone. Chest. 1993; 103: 628–629. 8432172 10.1378/chest.103.2.628

[42] Rezeig MA , Fashir BM . Biliary tract obstruction due to sarcoidosis: a case report. Am. J. Gastroenterol. 1997; 92: 527–528. 9068488

[43] Stitt RS , Greenwood R , Laczek J . A surprising cause of acute right upper quadrant pain. Case Rep. 2014; 2014: bcr2013201943. 10.1136/bcr-2013-201943 PMC4127761 25103316

[44] Masuda K , Takenaga S , Morikawa K , Kano A , Ojiri H . Hepatic sarcoidosis with atypical radiological manifestations: A case report. Radiol Case Rep. 2018; 13: 936–939. 30105085 10.1016/j.radcr.2018.06.013 PMC6077144

[45] Farouj NE , Cadranel JF , Mofredj A *et al*. Ductopenia related liver sarcoidosis. World J. Hepatol. 2011; 3: 170–174. 22509431 10.4254/wjh.v3.i6.170 PMC3326726

[46] Harder H , Büchler MW , Fröhlich B *et al*. Extrapulmonary sarcoidosis of liver and pancreas: a case report and review of literature. World J. Gastroenterol. 2007; 13: 2504–2509. 17552036 10.3748/wjg.v13.i17.2504 PMC4146771

[47] Tan CB , Rashid S , Rajan D , Gebre W , Mustacchia P . Hepatic sarcoidosis presenting as portal hypertension and liver cirrhosis: case report and review of the literature. Case Rep. Gastroenterol. 2012; 6: 183–189. 22679408 10.1159/000338355 PMC3364039

[48] Mueller S , Boehme MW , Hofmann WJ , Stremmel W . Extrapulmonary sarcoidosis primarily diagnosed in the liver. Scand. J. Gastroenterol. 2000; 35: 1003–1008. 11063165 10.1080/003655200750023110

[49] Sharma NK , Sherker AH . Corticosteroids for treatment of decompensated hepatic sarcoidosis: 744. Official J. Am. College Gastroenterol. 2006; 101: S303.

[50] Kothakota SR , Kumar Nair A , Sasidharan M , Kareem H , Praveen Kumar C , Kanala J . Extrapulmonary sarcoidosis manifested as cirrhosis with portal hypertension. Middle East J Dig Dis. 2021; 13: 160–162. 34712455 10.34172/mejdd.2021.220 PMC8531926

[51] Karagiannidis A , Karavalaki M , Koulaouzidis A . Hepatic sarcoidosis. Ann. Hepatol. 2006; 5: 251–256. 17151576

[52] Deliwala SS , Hussain M , Ponnapalli A *et al*. Sarcoidosis masquerading as long‐standing cholestasis. Gastroenterology Res. 2021; 14: 112–115. 34007353 10.14740/gr1360 PMC8110232

[53] Kremer JM, Alarcón GS, Lightfoot RW Jr, Willkens RF, Furst DE, Williams HJ, Dent PB, Weinblatt ME. Methotrexate for rheumatoid arthritis. Suggested guidelines for monitoring liver toxicity. American College of Rheumatology. Arthritis Rheum. 1994; 37: 316–28. 10.1002/art.1780370304 8129787

[54] Ørum M , Hilberg O , Krag S , Bendstrup E . Beneficial effect of infliximab on refractory sarcoidosis. Dan. Med. J. 2012; 59: A4535. 23290282

[55] Korsten P , Mirsaeidi M , Sweiss NJ . Nonsteroidal therapy of sarcoidosis. Curr. Opin. Pulm. Med. 2013; 19: 516–523. 23884295 10.1097/MCP.0b013e3283642ad0 PMC4134017

